# P2X7 receptor mediates the anti-tumor effect of running on cervical cancer in mice

**DOI:** 10.1007/s11302-026-10143-9

**Published:** 2026-04-15

**Authors:** Hai-Yun Xiong, Rui Li, Xiu-Min Hu, Yi Zhang, Patrizia Rubini, Peter Illes, Yong Tang

**Affiliations:** 1 International Joint Research Centre on Purinergic Signalling, School of Health and Rehabilitation, Chengdu University of Traditional Chinese Medicine, Chengdu, 611137 China; 2 Rudolf Boehm Institute for Pharmacology and Toxicology, University of Leipzig, Leipzig, 04107 Germany

**Keywords:** Running, P2X7 receptor (P2X7R), Cervical cancer, Anti-tumor effect

## Abstract

Previous studies have suggested that running exerts anti-tumor effects on various cancers through multiple pathways. It has been reported that the P2X7 receptor (P2X7R) may display anti-tumor activity in cervical cancer cells, and running can regulate P2X7R expression and function in mice. However, the specific impact of running on cervical cancer and whether its underlying mechanism is associated with the regulation of P2X7R levels remains to be elucidated. In this study, mice bearing U14 cervical cancer tumors were used as a model to explore the association between running and P2X7R in cervical cancer. The results showed that running significantly inhibited tumor progression in mice, accompanied by an increase in P2X7R protein expression in tumor tissues. Additionally, treatment with the P2X7R antagonist JNJ-47965567 alone or in combination with running intervention promoted tumor growth and attenuated the anti-tumor effect of running. In contrast, the P2X7R agonist BzATP alone or combined with running intervention exerted anti-tumor effects and enhanced the anti-tumor effect of running. In conclusion, the present findings suggest only a pharmacological association between P2X7R and the anti-tumor effect of running against cervical cancer progression, given that genetic validation was not performed in this study. The potential involvement of immune mechanisms remains an inference and requires further experimental validation. As a possible therapeutic target, P2X7R provides supportive data for the treatment of cervical cancer and the clinical exploration of running as an adjuvant intervention.

## Introduction

Cervical cancer is one of the most representative malignant tumors of the female reproductive system worldwide and consistently ranks fourth among common cancers in women globally [1]. In 2020, there were 604,000 new cases of cervical cancer worldwide, with over 340,000 deaths [2]. Although human papilloma virus (HPV) vaccines and cervical cytology screening techniques have been gradually promoted, issues like uneven vaccine distribution [3] and high treatment resistance in advanced cases [4] still limit the effectiveness of prevention and control, so it is urgent to explore safe and accessible auxiliary intervention measures to reduce the disease burden.

Running, as a low-cost, high-acceptance cancer intervention, has grown increasingly valuable in the field of cancer prevention and control in recent years. Epidemiological evidence clearly confirms a negative correlation between running and malignant tumor risk [5], while existing evidence also shows that regular running can reduce cancer recurrence risk [6] and improve the quality of life [7]. Running has also been proven to reduce the risk of HPV infection progressing to cervical precancerous lesions [8, 9], further highlighting its potential in cervical cancer prevention and control. From a mechanistic research perspective, existing evidence has revealed that running exerts anticancer effects through multiple pathways. First, it enhances the efficacy of clinical treatments such as radiotherapy [10] and chemotherapy [6, 11], providing synergistic support for anticancer therapy. Second, running regulates the tumor immune microenvironment via multiple key factors; for instance, it activates interleukin (IL)−15 to drive CD8⁺ T cells [12], produces adrenaline to induce IL-6 for natural killer (NK) cell mobilization [13], and triggers myocyte-derived miR-29a-3p to promote anti-tumor immunity [14]. Third, the serum of running individuals can directly inhibit cancer cell proliferation [15–17]. Among these, immune regulation is regarded as one of the core pathways through which running exerts its tumor-suppressive effects [14, 18]. This regulatory process often relies on the precise regulation of immune-related signaling molecules, and the purinergic signal receptor family is exactly the key molecular cluster that mediates the balance between immune function and the tumor microenvironment (TME) [19]. Among these receptors, the P2X7 receptor (P2X7R) plays a particularly prominent role [20, 21].

P2X7R is an ATP-gated ion channel [22] and is widely expressed in nearly all immune cells involved in anti-tumor immunity [20, 23, 24]. The role of P2X7R in anti-tumor responses is highly complex, and preclinical studies have shown that it exerts contradictory effects—either inhibiting or promoting tumor growth and metastasis [25–28]. P2X7R serves as a key regulator in epithelial tumors and mediates multiple cell death processes, including apoptosis, necrosis, and pyroptosis, at both cellular and organ levels. In colorectal cancer, P2X7R promotes angiogenesis and tumor-associated macrophage infiltration by activating the NF-κB signaling pathway [29]. In melanoma, P2X7R regulates the release of exosomes and microvesicles, thereby affecting apoptosis and necrosis as well as promoting tumor metastasis [30]. Furthermore, P2X7R induces apoptosis and pyroptosis by activating the NLRP3 inflammasome, which in turn promotes tumor cell proliferation, angiogenesis, invasion, and metastasis [31]. Existing studies have confirmed the anti-tumor effect of the P2X7R in cervical cancer [32], and running exerts a regulatory effect on P2X7R [33, 34]. However, the effect of running on cervical cancer remains unclear, and it is also unknown whether its underlying mechanism is related to the regulation of P2X7R levels in cervical cancer U14 tumor-bearing mice. Therefore, we aim to explore the impact of aerobic running on P2X7R expression and anti-tumor effects in cervical cancer mice.

## Materials and methods

### Cell culture and therapeutic agents

Mouse cervical cancer cell lines (U14 (Luc2); ATCC, Germany) were cultured in Dulbecco’s Modified Eagle’s Medium (DMEM, 11995065, Gibco, USA) supplemented with 10% fetal bovine serum (BC-SE-FBS01, Biochannel, China) and 1% penicillin–streptomycin (SV30010, Cytiva, China) at 37 °C in a 5% CO₂ incubator. U14 (Luc2) cells stably express luciferase, which enables in vivo imaging of the cells in xenograft animal models after their transplantation. For in vivo administration, BzATP (2′(3′)-O-(4-benzoylbenzoyl) adenosine 5′-triphosphate, B6396), a prototypic agonist of P2X7R, was purchased from Sigma (USA). BzATP was dissolved in normal saline. The selective P2X7 antagonist JNJ-47965567 was purchased from AbMole Biosciences (M6848, USA). JNJ-47965567 was first dissolved in DMSO up to a concentration of 100 mg/mL, and then formulated with 30% SBE-β-cyclodextrin to reach the final concentration [35]. Injections were administered alternately on both sides of the abdomen to minimize irritation. JNJ-47965567 is termed in the following only as JNJ throughout.

### Murine tumor models and treatment

Specific-pathogen-free female Kunming mice (KM, 5–6 weeks old, 20–22 g) were purchased from Chengdu Dashuo Laboratory Animal Co. (China). The mice were acclimatized to the environment for one week before the experiment. One day prior to modeling, hair at the injection site (right forelimb near the back) was removed using depilatory cream. U14 (Luc2) cells were resuscitated, cultured to logarithmic growth phase, digested with trypsin (Sigma), and collected. A single-cell suspension (1 × 10^7^/mL) was prepared with normal saline, and 0.2 mL (approximately 2 × 10^6^ viable cells) was subcutaneously injected into the right abdomen of the mice. Tumor volume and mouse body weight were recorded every 3 days using a vernier caliper after tumor inoculation. On day 16, tumor volume and metastasis in mice were recorded using an in vivo fluorescence imager (Tanon ABL X6, China) under resting conditions.

Tumor volume was calculated using a vernier caliper with the formula: tumor volume (mm^3^) = 1/2 × tumor length (mm) × (tumor width)^2^ (mm^2^). Tumor volume was measured by researchers blinded to group allocation. Randomization procedure: One day before modeling, all mice were consecutively numbered from 1 to n. Mice were randomly divided into 8 groups using a computer-generated random number table. Group allocation was performed by an independent researcher who was not involved in subsequent experiments and data collection to avoid selection bias. Before modeling, the following groups of mice were constituted: CON (control) group, RUN group, JNJ group, JNJ + RUN group, DMSO group, BzATP group, BzATP + RUN group, and NS (normal saline) group. On day 3 post-modeling, when the average tumor volume reached 60 mm^3^, tumor modeling was regarded as successful. On the day of modeling, running intervention was initiated in the RUN group, JNJ + RUN group, and BzATP + RUN group. One day before modeling, the JNJ group and JNJ + RUN group were pretreated with an intraperitoneal injection of JNJ (30 mg/kg, 200 μL) prior to tumor inoculation. JNJ was injected every 3 days after modeling. The DMSO group received an equal volume of DMSO and 30% SBE-β-cyclodextrin solution. One day before modeling, the BzATP group and BzATP + RUN group were pretreated with an intraperitoneal injection of BzATP (10 mg/kg, 200 μL) prior to tumor inoculation. BzATP was injected every 3 days after modeling. The NS group received an equal volume of normal saline.

### Running test and prescription

The running intervention in this study was forced treadmill running. According to guidelines established by the American College of Sports Medicine (ACSM) [36], cancer patients are recommended to run 3 to 5 days per week or more, with running intensity (measured by running speed) ranging from moderate to vigorous and each session lasting 30 to 60 min. Additionally, intensity should be defined based on an individual’s maximum tolerable running capacity, which is referred to as maximal oxygen uptake (VO₂max). Based on this, to determine an appropriate running training protocol, we conducted a maximal running test on KM mice [37]. The mice were made to run on a treadmill (JD-PT, Shanghai Jide Teaching Laboratory Factory, China) starting at a speed of 6 m/min, with the speed increased by 3 m/min every 3 min and a 0° incline, until they voluntarily exhausted themselves. Upon exhaustion, the mice were quickly removed from the treadmill, and the final speed (m/min) was recorded. Furthermore, studies have shown that moderate-intensity running in mice exerts anti-tumor effects [38]. Therefore, the running training protocol consisted of daily moderate-intensity running (16 m/min) for 30 min over 16 consecutive days. On day 17 (1 day after the end of the 16-day running training), the mice were anesthetized with 2% isoflurane inhalation. Under deep anesthesia, mice were sacrificed by cervical dislocation, and tumor tissues were dissected and collected. Humane endpoints were strictly applied in accordance with animal ethics guidelines. Mice were euthanized if tumor volume exceeded 1500 mm^3^, or if severe weight loss, impaired mobility, or obvious distress was observed.

### In vivo fluorescence imaging

On day 16 of modeling, 100 µL of Luc2 Substrate (CTCC-Luc-002, MeisenCTCC, China) was intraperitoneally injected into each mouse to trigger luciferase activity. After 10–15 min, the mice were imaged using an in vivo imaging system (Tanon ABL X6, China), and the acquired images were analyzed with image analysis software.

### Hematoxylin and eosin staining

Tumor tissues were fixed in 10% neutral buffered formalin (NBF) for 24–48 h, followed by gross morphological observation. After routine paraffin embedding, 4-μm-thick sections were prepared. The sections were deparaffinized, rehydrated, stained with hematoxylin (HE; 5–8 min) and eosin (1–2 min), then dehydrated, cleared, and mounted. Histological changes were observed under a bright-field microscope.

### Immunohistochemical (IHC) examination

Tumor tissues were fixed in 4% paraformaldehyde, embedded in paraffin, and sectioned into 5‑μm‑thick slices. The sections were then incubated with Ki67 primary antibody (1:2000, 28,074–1-AP, Proteintech, USA) at 4 °C overnight, followed by incubation with an HRP-conjugated secondary antibody kit (PV-9001, Zhongshan Jinqiao Biotechnology Co., Ltd., China) at room temperature for 1 h. After secondary antibody incubation, sections were developed with DAB chromogen kit (ZLI-9018, Zhongshan Jinqiao Biotechnology Co., Ltd., China), counterstained with hematoxylin (H9627, Sigma, USA) to visualize cell nuclei, dehydrated in a graded ethanol series, cleared in xylene, and finally mounted with a coverslip. Sections were observed under a light microscope (DM500, Leica, Germany) and imaged (DM1000, Leica, Germany). Image-Pro Plus was used to count total and positive cells and calculate the positive rate.

### Immunofluorescent staining assay

Tumor tissues were fixed in 4% paraformaldehyde solution and stored in a 4 °C refrigerator for 24 h. After the tumor tissues settled to the bottom, they were equilibrated in PBS containing 30% sucrose and stored in a 4 °C refrigerator until they completely sank; the PBS solution with 30% sucrose was replaced daily for 2 consecutive days. The tumor tissues were placed in embedding cassettes, embedded in OCT compound on a pre-cooled (−20 °C) platform, and then frozen in a pre-cooled microtome (CM1950, Leica, Germany). Twenty-micrometer-thick sections were stained with P2X7 Antibody (D-1) (1:50, sc-514962, Santa Cruz Biotechnology, USA). After primary antibody incubation, sections were mounted with fluorescence anti-fade mounting medium containing DAPI (S2110, Beyotime, China) to visualize cell nuclei. A laser confocal microscope (N6400300, Olympus, Japan) was used to observe and capture images, and the Image-Pro Plus software was used to process the captured immunofluorescence images.

### Western blot

Total proteins were extracted from tumor tissues using lysis buffer (FNN0071, Invitrogen, USA) supplemented with a protease inhibitor (4693159001, Roche, Switzerland). The resulting supernatant was quantified using a Pierce BCA Protein Assay Kit (23227, Thermo Scientific, USA). A total of 30 μg of protein was separated via 10% SDS-PAGE gel (NP0301BOX, Invitrogen, USA) and then transferred onto a PVDF membrane (IPVH00010, Immobilon, Germany). The PVDF membrane was blocked with 5% non-fat milk at room temperature for 1 h, followed by incubation with primary antibodies against P2X7 Antibody (D-1) (1:500, sc-514962, Santa Cruz, USA) and β-actin (1:5000, GB15003-100, Servicebio, China) at 4 °C overnight. After washing with TBST, the membrane was incubated with species-specific HRP-conjugated secondary antibodies (1:5000; goat anti-rabbit IgG, SA00013-4; goat anti-mouse IgG, SA00013-1; Proteintech, USA) at room temperature for 1 h. Finally, a chemiluminescence system (BLT GelView 5000Plus, Guangzhou Biolight Biotechnology, China) was used to detect the protein levels.

### Statistical analysis

All data were presented as mean ± SEM and processed using GraphPad Prism 9.0. The Shapiro–Wilk test was used to verify data normality. Unpaired Student’s *t*-test was applied for comparisons between two groups, while one-way ANOVA with Tukey’s post hoc test was used for comparisons among three or more groups, and two-way ANOVA with Tukey’s post hoc test was used for comparisons involving two independent variables. A two-tailed *p* < 0.05 was considered statistically significant. All experiments were independently repeated at least 3 times to ensure reproducibility.

## Results

### The inhibitory effect of running on cervical cancer

**Fig. 1:**
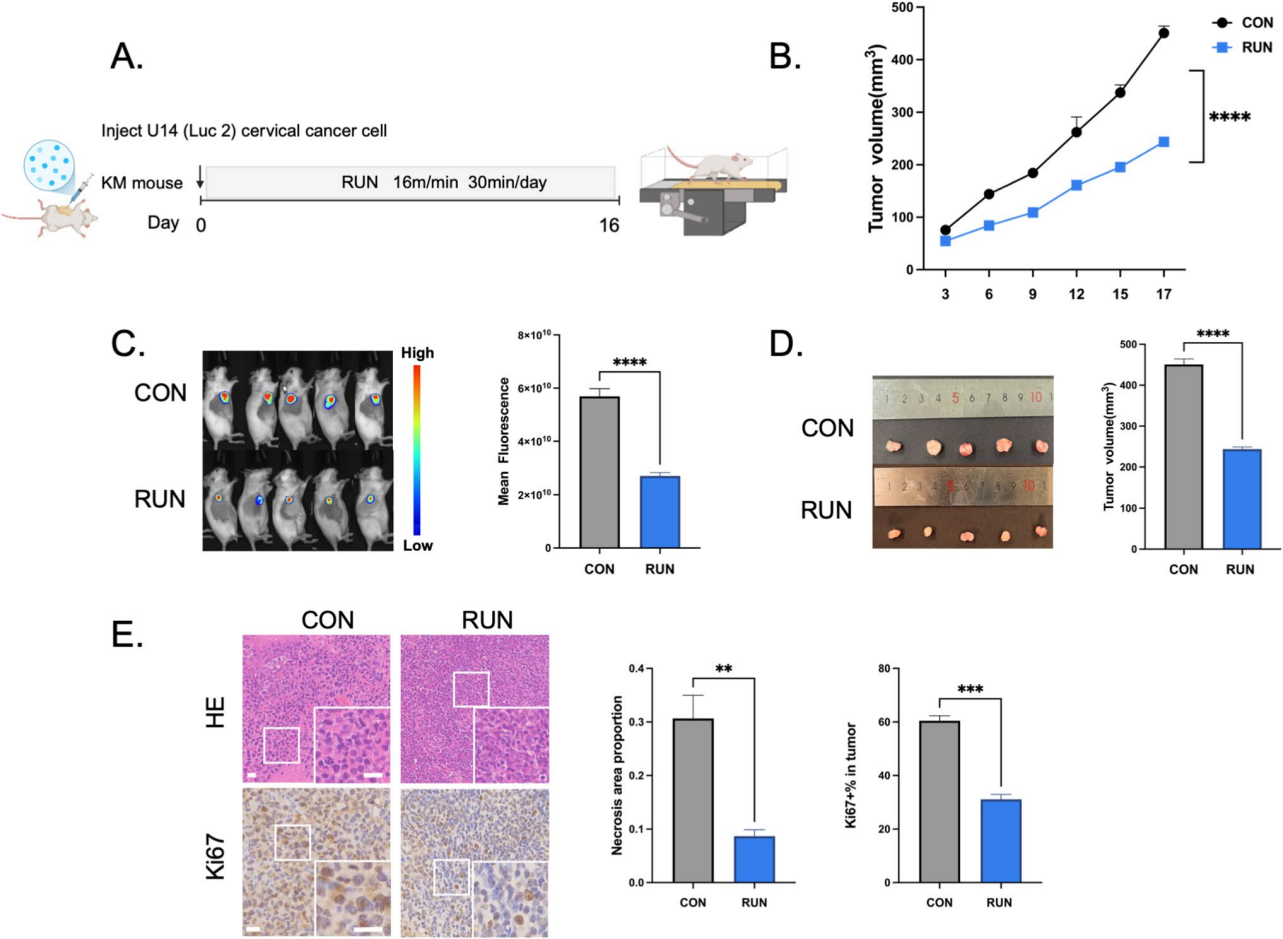
The inhibitory effect of running on cervical cancer. **A** Schematic diagram of the experimental design. Female Kunming (KM) mice bearing U14 (Luc2) cervical cancer cells were randomly divided into two groups: the control (CON) group and the running (RUN) group. On the day of modeling, mice in the RUN group received moderate-intensity running (speed: 16 m/min, 30 min per day) for 16 consecutive days; mice in the CON group remained sedentary without running training. On the 17th day, all mice were anesthetized with isoflurane, and tumor tissues were collected. **B** Tumor growth curves of cervical cancer-bearing mice within 17 days (*n* = 6, two-way ANOVA). **C** In vivo whole-body fluorescence images of mice on the 16th day after U14 (Luc2) cell inoculation. The fluorescence signal at the tumor site in mice of the RUN group was significantly weaker than that in the CON group, suggesting that running can inhibit cervical cancer growth (*n* = 6, Student’s *t*-test). **D** Representative images of harvested tumors (left panel) and quantitative analysis of final tumor volumes (right panel) on day 17 (*n* = 6, Student’s *t*-test). **E** Representative hematoxylin and eosin (HE)-stained images of tumor tissues (upper left panel; scale bar: 20 μm) and quantitative analysis of tumor necrosis areas (right panel; *n* = 3, Student’s *t*-test). Representative Ki67 immunohistochemistry (IHC)-stained images of tumor tissues (lower left panel; scale bar: 20 μm) and quantitative analysis of Ki67-positive cell rates (right panel; *n* = 3, Student’s *t*-test). All data are expressed as mean ± standard error of the mean (SEM). *p < 0.05, **p < 0.01, ***p < 0.001, ****p < 0.0001

We aimed to investigate the inhibitory effect of running on cervical cancer. Prior to the formal intervention, a graded running test was conducted on KM mice, and their maximum running speed was finally determined to be 32 m/min, providing a reference for setting the running intensity in subsequent formal experiments. Cervical cancer-bearing mouse models were established by injecting U14 (Luc2) cervical cancer cells. On the day of modeling, mice were randomly divided into the CON group and the RUN group. On the day of modeling, mice in the RUN group started daily moderate-intensity running on a treadmill at 16 m/min for 30 min per day, for 16 consecutive days; mice in the CON group received no running training (Fig. 1A). On the 17th day, tumor growth in both groups was evaluated. Results showed that compared with the CON group, the RUN group exhibited a significantly lower tumor growth rate and a markedly reduced tumor volume (Fig. 1B, C, D). HE staining analysis revealed that the proportion of necrotic areas in tumor tissues in the RUN group was significantly lower than that in the CON group (Fig. 1E). Immunohistochemical detection of Ki67 protein expression in the tumor microenvironment showed that the positive expression rate of Ki67 in tumor cells of the RUN group was significantly lower than that in the CON group (Fig. 1E), indicating that running can effectively inhibit the proliferation of tumor cells in cervical cancer-bearing mice.

### The effect of running intervention on P2X7R expression level in tumor tissues

**Fig. 2:**
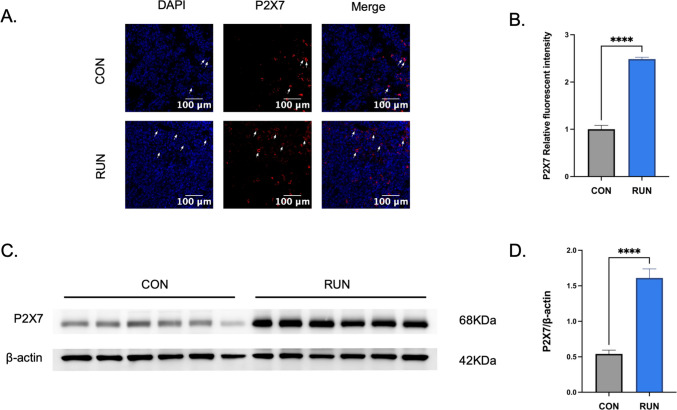
The effect of running intervention on P2X7R expression level in tumor tissues. **A** Immunofluorescence (IF) staining of P2X7R in tumor tissues (scale bar: 100 μm). **B** Quantitative analysis of P2X7R expression in tumor tissues (*n* = 3, Student’s *t*-test). **C** Western blotting images of P2X7R in tumor tissues. **D** Quantitative analysis of P2X7R expression intensity (*n* = 6, Student’s *t*-test). All data are expressed as mean ± SEM. *p < 0.05, **p < 0.01, ***p < 0.001, ****p < 0.0001

Next, we investigated whether the P2X7R is associated with the inhibitory effect of running on cervical cancer. First, immunofluorescence results further showed that the fluorescence signal intensity of the P2X7R in tumor tissues of the RUN group was higher than that in the CON group (Fig. 2A, B). Subsequently, Western Blotting results showed that the expression level of P2X7R protein in tumor tissues of the RUN group was significantly higher than that in the CON group (Fig. 2C, D). These data indicate that running upregulates P2X7R protein expression in tumor tissues.

### JNJ promotes tumor progression and attenuates running-induced anticancer effects

**Fig. 3:**
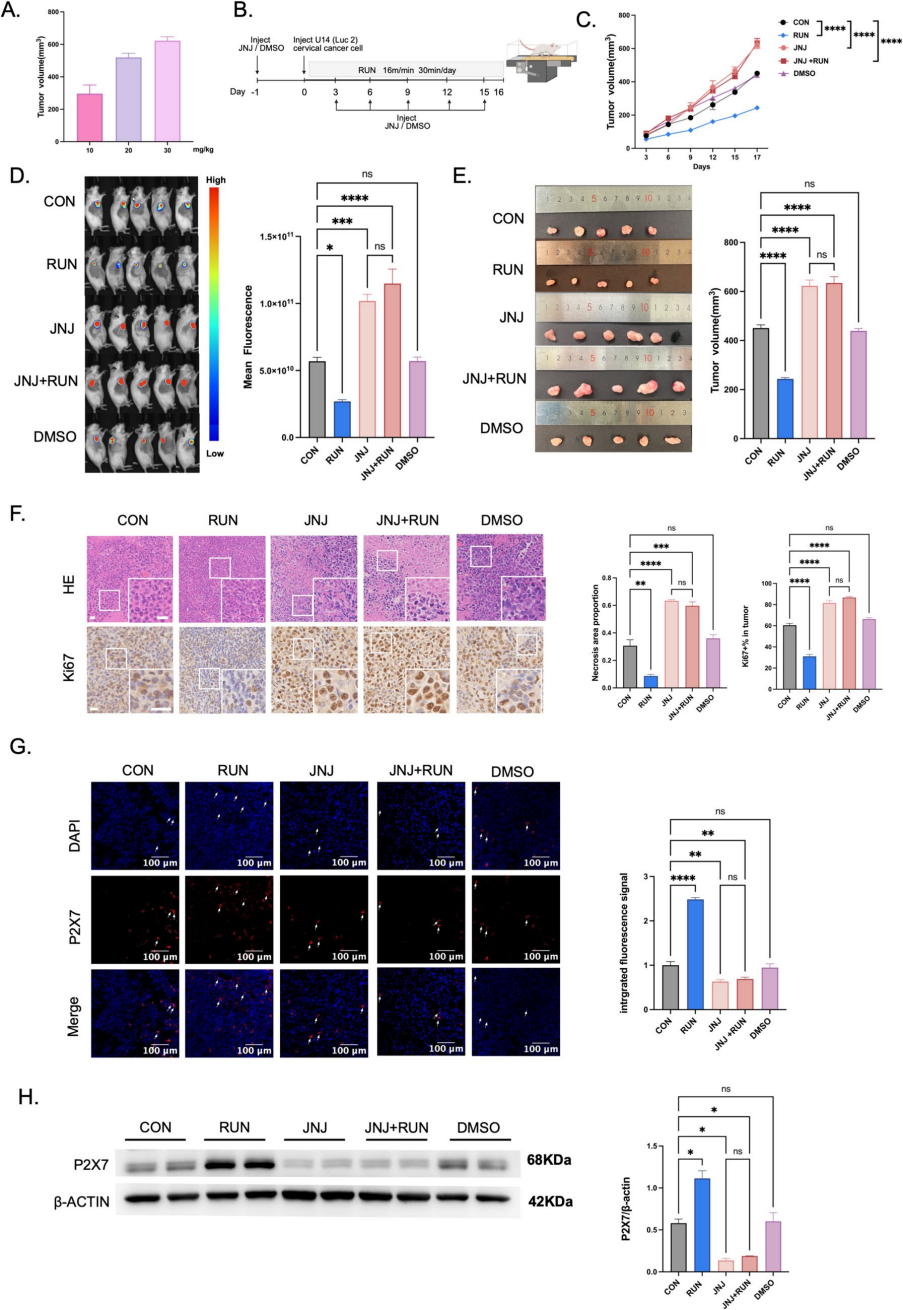
JNJ promotes tumor progression and reverses running-induced anticancer effects. **A** JNJ dose screening for tumor promotion: Cervical cancer-bearing mice were treated with JNJ (10, 20, 30 mg/kg) 1 day pre-modeling and injected every 3 days post-modeling; tumors were collected on day 17 (*n* = 6). For the following experiments a 30 mg/kg dose of JNJ was chosen. **B** Experimental design: Female KM mice were divided into JNJ, JNJ + RUN, and DMSO groups 1 day pre-modeling. Injections of JNJ (JNJ and JNJ + RUN groups) or DMSO (DMSO group) were administered starting 1 day pre-modeling and continued every 3 days post-modeling. On the day of modeling, the JNJ + RUN group received moderate running (16 m/min, 30 min/session, 16 days). **C** Tumor growth curves of cervical cancer-bearing mice over 17 days (*n* = 6, two-way ANOVA). **D** In vivo whole-body fluorescence images of mice on day 16 post-U14 (Luc2) cell inoculation (*n* = 6, one-way ANOVA). **E** Harvested tumor images (left panel) and final tumor volume quantification (right panel) on day 17 (*n* = 6, one-way ANOVA). **F** HE staining (upper left panel; scale bar: 20 μm) and tumor necrosis quantification (middle panel; *n* = 3, one-way ANOVA). Ki67 staining (lower left panel; scale bar: 20 μm) and Ki67-positive cell rate quantification (right panel; *n* = 3, one-way ANOVA). **G** P2X7R immunofluorescence in tumor tissues (left panel; scale bar: 100 μm) and quantification of the immunofluorescence (right panel; *n* = 3, one-way ANOVA). **H** Western blotting for P2X7R in tumor tissues (left panel) and quantification of the blotting (right panel; *n* = 6, one-way ANOVA). All data are expressed as mean ± SEM. *p < 0.05, **p < 0.01, ***p < 0.001, ****p < 0.0001, ns = not significant

To investigate whether P2X7R is functionally involved in the anti-tumor effects of running against cervical cancer, we conducted in vivo intervention experiments using JNJ, a specific P2X7R functional antagonist. First, we screened for the optimal concentration of JNJ: cervical cancer-bearing mice were treated with three concentration gradients (10 mg/kg, 20 mg/kg, and 30 mg/kg) (Fig. 3A). Tumor samples were collected and analyzed on day 17 after modeling, which showed that 30 mg/kg JNJ most significantly promoted cervical cancer growth, so this concentration was used in subsequent experiments. One day before cervical cancer modeling, mice were randomly divided into three groups: JNJ group, JNJ + RUN group, and DMSO group (solvent control). The JNJ and JNJ + RUN groups received intraperitoneal injections of 200 μL JNJ solution (30 mg/kg), while the DMSO group received an equal volume of 200 μL DMSO solvent. The same injections were repeated every 3 days after modeling. Additionally, the JNJ + RUN group underwent daily treadmill running (16 m/min, 30 min/session, for 16 consecutive days), while the JNJ and DMSO groups received no running training with other feeding conditions kept consistent (Fig. 3B). Post-intervention tests revealed that JNJ alone (JNJ group) significantly promoted tumor growth in cervical cancer-bearing mice; when JNJ was combined with running (JNJ + RUN group), the inhibitory effect of running on cervical cancer was significantly attenuated (Fig. 3C, D, E). These results show that JNJ-mediated functional inhibition of P2X7R correlates with enhanced in vivo growth ability of cervical cancer and attenuated anti-tumor effect of running on cervical cancer, with pharmacological modulation supporting a possible role for P2X7R signaling in the anti-tumor effect of running. Notably, genetic validation of P2X7R involvement was not performed, and the evidence for P2X7R requirement is limited to a pharmacological sense.

The proportion of necrotic areas in tumor tissues was significantly higher in both the JNJ group and JNJ + RUN group than in the CON group (Fig. 3F). The Ki67-positive expression rate of tumor cells in the JNJ group and JNJ + RUN group was also significantly higher than that in the CON group, suggesting that JNJ is associated with enhanced tumor cell proliferation (Fig. 3F). Results of P2X7R immunofluorescence staining revealed that the fluorescence signal intensity of P2X7R in tumor tissues of the JNJ group and JNJ + RUN group was significantly lower than that in the CON group (Fig. 3G). Western blotting analysis of P2X7R protein expression in tumor tissues showed that the relative expression level of P2X7R protein in the JNJ group and JNJ + RUN group was significantly lower than that in the CON group, demonstrating that JNJ treatment was associated with reduced P2X7R protein expression in tumor tissues (Fig. 3H).

### BzATP inhibits tumor progression and enhances running-induced anticancer effects

**Fig. 4:**
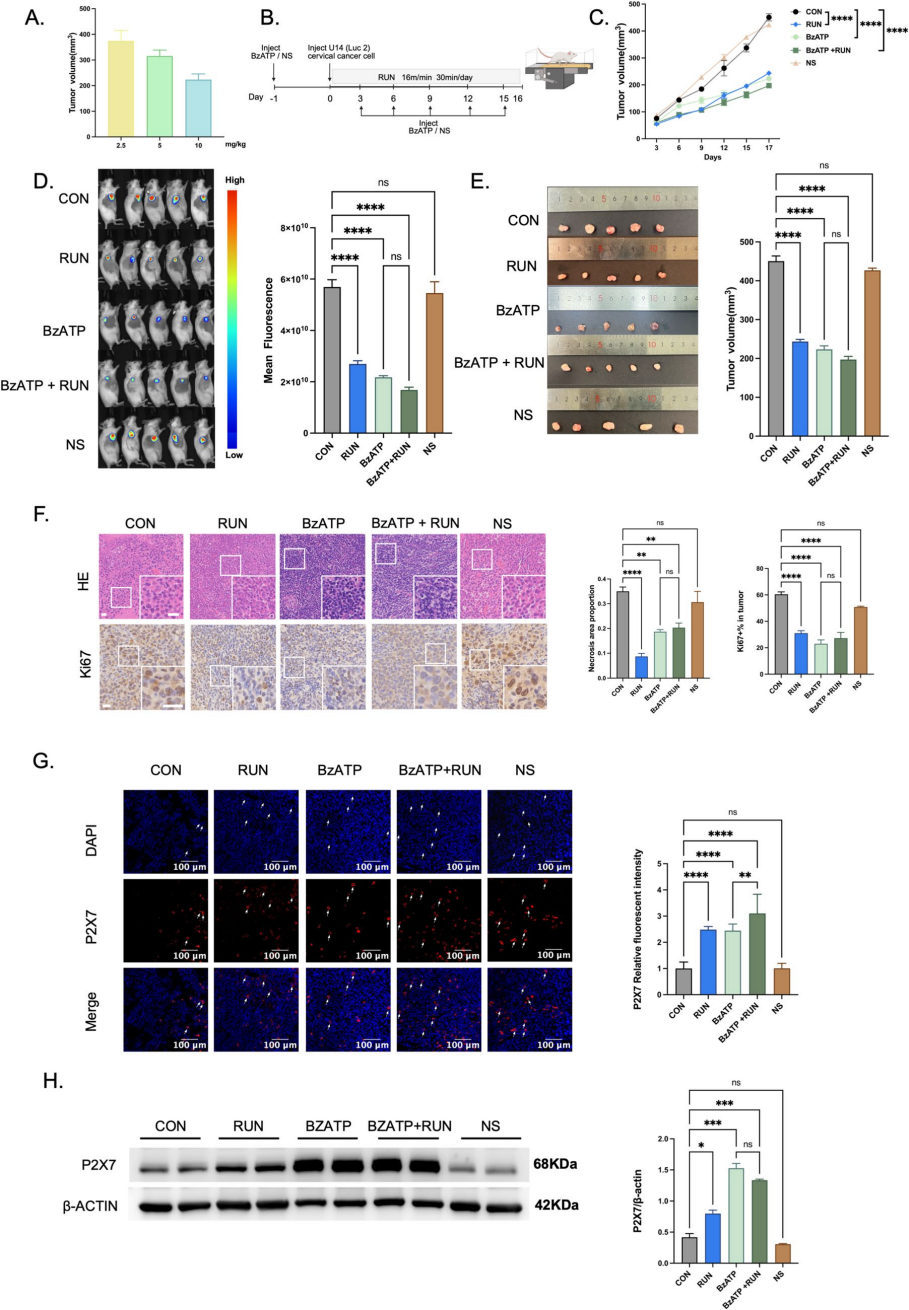
BzATP inhibits tumor progression and enhances running-induced anticancer effects. **A** Screening of optimal BzATP intervention doses: three doses (2.5, 5, 10 mg/kg) were used, and tumor size was measured on day 17 post-modeling (*n* = 6). **B** Experimental design: Female KM mice were divided into BzATP, BzATP + RUN, and NS groups at 1 day pre-modeling. Injections of BzATP (BzATP and BzATP + RUN groups) or normal saline (NS group) were given starting at 1 day pre-modeling and then repeated every 3 days post-modeling. On the day of modeling, the BzATP + RUN group received moderate running (16 m/min, 30 min/session, 16 days). **C** Tumor growth curves of cervical cancer-bearing mice over 17 days (*n* = 6, two-way ANOVA). **D** In vivo whole-body fluorescence images of mice on day 16 post-U14 (Luc2) cell inoculation (left panel) and quantitative evaluation (right panel; *n* = 6, one-way ANOVA). **E** Harvested tumor images (left panel) and final tumor volume quantification (right panel) on day 17 (*n* = 6, one-way ANOVA). **F** HE staining (upper left panel; scale bar: 20 μm) and tumor necrosis quantification (middle panel; *n* = 3, one-way ANOVA). Ki67 staining (lower left panel; scale bar: 20 μm) and Ki67-positive cell rate quantification (right panel; *n* = 3, one-way ANOVA). **G** P2X7R immunofluorescence in tumor tissues (left panel; scale bar: 100 μm) and quantification of the immunofluorescence (right panel; *n* = 3, one-way ANOVA). **H** Western blotting for P2X7R in tumor tissues (left panel) and quantification of the blotting (right panel; *n* = 6, one-way ANOVA). All data are expressed as mean ± SEM. *p < 0.05, **p < 0.01, ***p < 0.001, ****p < 0.0001, ns = not significant

To investigate the potential association of P2X7R signaling with cervical cancer growth, we used BzATP as a specific P2X7R activator. To screen for the optimal BzATP intervention concentration, three concentration gradients (2.5 mg/kg, 5 mg/kg, and 10 mg/kg) were set. Tumor size was measured on day 17 after modeling, and results showed that the 10 mg/kg concentration exerted the most significant tumor-inhibitory effect; thus, this concentration was used in all subsequent experiments (Fig. 4A). One day before tumor modeling, experimental mice were randomly divided into three groups: BzATP group, BzATP + RUN group, and NS group. Interventions were then administered: the BzATP and BzATP + RUN groups received an intraperitoneal injection of 200 μL BzATP solution (10 mg/kg), while the NS group received an intraperitoneal injection of 200 μL normal saline. After modeling, the above group-specific injection protocol was repeated every 3 days. Meanwhile, mice in the BzATP + RUN group underwent daily running intervention (16 m/min, 30 min/day, for 16 consecutive days), whereas mice in the BzATP and NS groups received no running training (Fig. 4B). After the intervention period, tumor growth indices of mice in each group were detected and analyzed. Results showed that single BzATP intervention (BzATP group) significantly inhibited tumor growth in cervical cancer-bearing mice; when BzATP was combined with running training (BzATP + RUN group), the anti-tumor effect on cervical cancer was further enhanced compared with single BzATP intervention (Fig. 4C, D, E). These results suggest that P2X7R signaling is associated with inhibited cervical cancer growth and can produce a synergistic anti-tumor effect with running, based on pharmacological evidence.

Tumor tissues were subjected to HE staining, and necrotic areas were quantitatively analyzed. In the BzATP group and BzATP + RUN group, the proportion of tumor necrotic areas was significantly lower than that in the CON group. This finding indicates that BzATP intervention significantly reduced the degree of tumor necrosis (Fig. 4F). In the detection of tumor cell proliferation activity, results of the positive expression rate of Ki67, a cell proliferation marker, showed that the rates in the BzATP group and BzATP + RUN group were significantly lower than those in the CON group, suggesting that BzATP is associated with reduced tumor cell proliferation (Fig. 4F). Results of the P2X7R immunofluorescence staining showed that the fluorescence signal intensity of P2X7R in tumor tissues of the BzATP group and BzATP + RUN group was significantly stronger than that in the CON group (Fig. 4G). In addition, when detecting the expression level of P2X7R protein in tumor tissues via Western blotting, it was found that the relative expression of P2X7R protein in the BzATP group and BzATP + RUN group was also significantly higher than that in the CON group (Fig. 4H). These results indicate that BzATP treatment was associated with upregulated P2X7R protein expression in tumor tissues.

## Discussion

In this study, we demonstrated that running inhibits tumor progression in mice bearing cervical cancer, and increased P2X7R protein expression was observed in tumor tissues. When the P2X7R functional antagonist JNJ was used alone or in combination with running intervention, both treatments promoted the growth of cervical cancer tumors and attenuated the anti-tumor effect of running. Notably, when the P2X7R agonist BzATP was used alone or in combination with running intervention, both applications exerted anti-tumor effects, and the anti-tumor effect of running was enhanced. These observations indicate a pharmacological association between P2X7R signaling and the anti-tumor actions of running in cervical cancer, in the absence of genetic validation.

For decades, running has been associated with reduced cancer risk and delayed cancer progression [6, 8]. Such findings have been reported in several mouse tumor models, including the MCa-M3C breast cancer mouse model [38], B16F10 melanoma mouse model [13], Lewis lung cancer mouse model [14], and CT-26 colorectal cancer mouse model [39]. As the fourth most common cancer among women, cervical cancer imposes an epidemiological burden that has become a major public health issue threatening women’s health. However, no preclinical studies have explored the role of running in murine cervical cancer models. Therefore, we selected the U14 cervical cancer tumor-bearing mouse model for our research. The guidelines of the American College of Sports Medicine (ACSM) [36] recommend that cancer patients engage in moderate-to-high intensity running. Additionally, relevant preclinical studies have shown that moderate-intensity running may exert an inhibitory effect on tumors [37, 38]. In this study, we first conducted a maximal running test on KM mice and determined their maximal speed to be 32 m/min, and then defined moderate intensity as 50% of this maximum speed (16 m/min). This running intensity matches the proven intensity range in other tumor models (e.g., the MCa-M3C breast cancer mouse model [38]), highlighting the methodological reliability of the experimental results. The experimental results showed that moderate-intensity running inhibited tumor growth in U14 cervical cancer-bearing mice. These findings reveal a link between running dosage and tumor growth inhibition. Thus, when promoting running as an intervention for cancer survivors in subsequent practice, it is necessary to develop appropriate running prescriptions based on their individual conditions.

In this study, we observed increased P2X7R protein expression in tumor tissues after running. These findings complement existing reports on the regulation of P2X7R protein expression by running in oncological research [33, 34]. Previous studies have mostly focused on the regulatory effect of running on P2X7R protein expression in normal tissues (e.g., cardiac tissue [34, 40]). Conversely, this study is the first to show that running upregulates P2X7R protein expression in tumor tissues.

Previous studies have shown conflicting effects of P2X7R on tumor growth [25–28]: its activation promotes tumor proliferation and invasion in gastric cancer [41], whereas it effectively inhibits tumor growth when activated in non-small cell lung cancer (NSCLC) [42]. This discrepancy may be closely associated with tumor type and the complexity of experimental systems. For instance, in cervical cancer cell in vitro experiments, both the P2X7R agonist BzATP and antagonist JNJ have been shown to exert anti-tumor effects [32]. This finding is in line with the bidirectional functional characteristic of P2X7R. However, in the in vivo experiments of the present study using U14 cervical cancer tumor-bearing mice, only the anti-tumor effect of BzATP was consistent with the results of cervical cancer in vitro experiments, while JNJ-47965567 (JNJ) was associated with tumor growth. These observations suggest a potential role for the tumor microenvironment in the observed effects, although direct experimental evidence for immune modulation is lacking in the present study.

On one hand, the tumor microenvironment (TME) contributes to reshaping the functional orientation of P2X7R. In vitro experiments only focus on tumor cells as a single target, while in vivo tumor growth relies on a TME network composed of multiple factors, including immune cell infiltration and cytokine regulation. Since P2X7R is expressed in nearly all immune cells and most tumor cells [21], it is tempting to hypothesize that its activation in immune cells could contribute to enhanced antitumor immune responses, but this remains to be experimentally demonstrated.

On the other hand, the concentration of the P2X7R functional antagonist JNJ-47965567 used in this study was 30 mg/kg. This dose was selected based on our own preliminary experiments (see Fig. 3A) and previous reports demonstrating effective central nervous system penetration and target engagement in rodents [35, 43], but it has not been previously applied in tumor-related experiments.

Of note, this dose is higher than those of other P2X7R antagonists (such as AZ10606120 at 2 mg/kg) that have been associated with tumor-inhibitory effects in oncology studies [44]. Notably, the use of a relatively high dose of JNJ-47965567 (30 mg/kg i.p.) represents a limitation of the present study. Several potential explanations exist for the observed tumor-promoting effect. This high dose may exert non-specific or off-target effects that suppress anti-tumor immune surveillance. For example, it may directly affect the function of immune cells that are critical for anti-tumor immunity, such as macrophages and T cells, thereby indirectly promoting tumor growth. These possibilities represent important alternative explanations for the present results.

In addition, JNJ-47965567 was administered one day before tumor inoculation. This treatment schedule may have affected early tumor engraftment and establishment, rather than only later tumor progression. We cannot rule out that early P2X7R blockade modified the local microenvironment to favor tumor seeding and initial survival. Future studies with a lower-dose antagonist group and delayed-start protocol might distinguish effects on tumor engraftment from those on tumor growth.

The relatively high concentration of JNJ used in this study may be associated with P2X7R signaling in tumor cells**.** We hypothesize that this dose could also influence immune cell functions related to P2X7R signaling, and that such indirect effects on the immune microenvironment might outweigh direct effects on tumor cells, ultimately leading to a tumor-promoting outcome. However, these assumptions are not supported by direct immune profiling in the current study.

It is important to emphasize that tumor necrosis arises from multiple pathological mechanisms and cannot be used as a sole indicator of antitumor activity. Tumor necrosis is not induced solely by antitumor effects, but may be driven by pathological factors including rapid tumor growth, local hypoxia, and insufficient vascular supply. Thus, tumor necrosis should be interpreted cautiously and evaluated comprehensively together with tumor volume, Ki-67 proliferation index, and other histopathological features to assess tumor burden and progression more objectively. Accordingly, this study employed a multi-indicator comprehensive analysis to evaluate tumor progression, which avoids the bias of relying on tumor necrosis alone and ensures the reliability of the conclusions.

The present study demonstrated that aerobic running significantly upregulated P2X7R protein expression in tumor tissues of cervical cancer-bearing mice. Pharmacological interventions further supported that P2X7R signaling contributed to running-mediated anti-tumor effects. However, changes in protein expression alone did not confirm direct activation of P2X7R by running. Therefore, the detailed mechanisms underlying the anti-tumor effects of running remain to be further elucidated. These mechanisms may involve apoptosis, pyroptosis, inflammatory responses, and anti-tumor immunity. This study has several limitations. We did not explore the expression and role of specific splice variants. Previous studies have confirmed that aberrant P2X7R expression is closely associated with tumor progression, chemoresistance, and immune microenvironment remodeling [20, 27, 45]. However, most studies focus on the overall function of P2X7R. The role of specific subvariants remains unclear. The detailed mechanisms and potential involvement of specific subvariants of the receptor in running-induced anti-tumor effects have not been fully explored.

Future studies will focus on specific P2X7R subvariants and their expression patterns to further clarify their roles in exercise‑induced anti‑tumor effects. Future studies using P2X7R knockout mice will be necessary to genetically validate these findings. Further research will also be required to clarify the signaling pathways through which running regulates P2X7R protein expression and function. In addition, detailed immune characterization should be performed in future work to verify the above hypotheses. This may include immune cell populations and key cytokines, such as NK cells, CD8⁺ T cells, and macrophages. Future studies are warranted to evaluate vascularization and hypoxia using markers such as CD31 and HIF-1α. These will clarify the mechanisms of tumor necrosis and its association with tumor growth.

## Conclusions

This study demonstrated that running can inhibit tumor progression in U14 cervical cancer-bearing mice and upregulate the expression of P2X7R in tumor tissues. When the P2X7R functional antagonist JNJ was used alone or in combination with the running intervention, it promoted tumor growth and attenuated the anti-tumor effect of running; in contrast, when the P2X7R agonist BzATP was used alone or in combination with running, it exerted an anti-tumor effect and strengthened the anti-tumor effect of running. In conclusion, these observations are consistent with a pharmacological involvement of P2X7R in the anti-tumor actions of running in U14 cervical cancer-bearing mice, in the absence of genetic validation.

## Data Availability

Data is made available upon reasonable request.
